# Realization of a Type‐II Nodal‐Line Semimetal in Mg_3_Bi_2_

**DOI:** 10.1002/advs.201800897

**Published:** 2018-11-28

**Authors:** Tay‐Rong Chang, Ivo Pletikosic, Tai Kong, Guang Bian, Angus Huang, Jonathan Denlinger, Satya K. Kushwaha, Boris Sinkovic, Horny‐Tay Jeng, Tonica Valla, Weiwei Xie, Robert J. Cava

**Affiliations:** ^1^ Department of Physics National Cheng Kung University Tainan 701 Taiwan; ^2^ Department of Physics Princeton University Princeton NJ 08544 USA; ^3^ Department of Chemistry Princeton University Princeton NJ 08544 USA; ^4^ Department of Physics and Astronomy University of Missouri Columbia MO 65211 USA; ^5^ Department of Physics National Tsing Hua University Hsinchu 30013 Taiwan; ^6^ The Advanced Light Source Lawrence Berkeley National Laboratory Berkeley CA 94720 USA; ^7^ Department of Physics University of Connecticut Storrs CT 06269 USA; ^8^ Institute of Physics Academia Sinica Taipei 11529 Taiwan; ^9^ Physics Division National Center for Theoretical Sciences Hsinchu 30013 Taiwan; ^10^ Condensed Matter Physics and Materials Science Brookhaven National Laboratory Upton NY 11973 USA; ^11^ Department of Chemistry Louisiana State University Baton Rouge LA 70803 USA

**Keywords:** angle‐resolved photoemission spectroscopy, density functional theory calculations, Mg_3_Bi_2_, topological materials, topological semimetals

## Abstract

Nodal‐line semimetals (NLSs) represent a new type of topological semimetallic phase beyond Weyl and Dirac semimetals in the sense that they host closed loops or open curves of band degeneracies in the Brillouin zone. Parallel to the classification of type‐I and type‐II Weyl semimetals, there are two types of NLSs. The type‐I NLS phase has been proposed and realized in many compounds, whereas the exotic type‐II NLS phase that strongly violates Lorentz symmetry has remained elusive. First‐principles calculations show that Mg_3_Bi_2_ is a material candidate for the type‐II NLS. The band crossing is close to the Fermi level and exhibits the type‐II nature of the nodal line in this material. Spin–orbit coupling generates only a small energy gap (≈35 meV) at the nodal points and does not negate the band dispersion of Mg_3_Bi_2_ that yields the type‐II nodal line. Based on this prediction, Mg_3_Bi_2_ single crystals are synthesized and the presence of the type‐II nodal lines in the material is confirmed. The angle‐resolved photoemission spectroscopy measurements agree well with the first‐principles results below the Fermi level and thus strongly suggest Mg_3_Bi_2_ as an ideal material platform for studying the as‐yet unstudied properties of type‐II nodal‐line semimetals.

The low‐energy quasiparticles in semimetallic solid‐state systems provide an ideal platform for studying some of the rich physics of elementary particles that has been theoretically proposed but never observed.^1^, ^2^ The discovery of massless Weyl quasiparticles in Weyl semimetals represents a triumph of this approach.^3^, ^4^, ^5^, ^6^, ^7^, ^8^, ^9^ The exotic properties of Weyl semimetals such as the chiral anomaly and nonlocal transport have stimulated significant research interest in the topological semimetallic phases of condensed matter. Recently, a new direction of thinking has emerged—to search for new topological quasiparticles that have no counterparts in high‐energy physics. This idea offers the possibility for new topological phenomena that are not limited by the fundamental constraints that exist in high‐energy physics. A prominent example of this idea is the prediction and realization of type‐II Weyl fermions.^10^, ^11^, ^12^, ^13^, ^14^, ^15^ The energy–wavevector dispersion of Weyl quasiparticles in type‐II Weyl semimetals is tilted along a given direction. This tilting explicitly breaks Lorentz invariance, which is strictly required in high‐energy physics, and gives rise to many distinctive properties such as anisotropic negative magnetoresistance^16^, ^17^ and the existence of tilted surface bands.^18^

Another novel topological solid‐state phase is the nodal‐line semimetal in which the intersection of conduction and valence bands consists of 1D open or closed lines.^19^, ^20^, ^21^, ^22^, ^23^ In contrast to the massless electrons of Weyl semimetals, the low‐energy excitations of nodal‐line semimetals are very massive along the direction tangent to the nodal lines and massless along the two transverse directions in momentum space. There appears to be no counterpart of particles with extremely anisotropic mass in high‐energy physics. This peculiar bulk band dispersion of nodal‐line semimetals makes the density of states (DOS) of the low‐energy bulk excitations proportional to |*E* − *E*_F_|, in contrast with the (*E* − *E*_F_)^2^‐like DOS seen in Weyl semimetals. Thus, stronger electron correlation effects are expected in nodal‐line semimetals due to the higher DOS at the Fermi energy.

The nodal‐line band structure is topological in the sense that the winding number along a little loop that interlinks the line nodes is nonzero. The nontrivial bulk band topology of nodal‐line semimetals is accompanied by a 2D surface band whose dispersion resembles a flat drumhead surface.^19^, ^20^, ^24^ The type‐II Weyl and nodal‐line semimetallic phases have been realized in many compounds such as WTe_2_,^10^, ^25^ Ca_3_P_2_,^26^ Cu_3_PdN,^27^, ^28^ and PbTaSe_2_.^24^, ^29^ Combining the ideas of type‐II Weyl and nodal‐line semimetals leads to a new topological semimetallic phase named type‐II nodal‐line semimetal.^30^, ^31^ In type‐II nodal‐line semimetals, the linear dispersion at every point of the nodal line is strongly tilted along one transverse direction in a similar way as in type‐II Weyl semimetals, leading to remarkable differences in magnetic, optical, and transport properties compared with conventional nodal‐line semimetals.^30^ K_4_P_3_ is the first compound that has been theoretically proposed as a type‐II nodal‐line semimetal but there remains a lack of experimental evidence for its actual character. Moreover, K_4_P_3_ is extremely air‐sensitive, such that performing characterization experiments and manufacturing devices are very challenging. Very recently, Zhang et al. theoretically proposed Mg_3_Bi_2_ to be a type‐II nodal‐line semimetal in the absence of spin–orbit coupling (SOC) and that it becomes a Dirac semimetal when SOC is included, but experimental evidence is lacking.^32^ Therefore, there is a pressing need for the realization of an experimental viable type‐II nodal‐line semimetal that is stable enough to enable the study of the expected and unexpected exotic behaviors of this new type of topological matter.

In this work, we first show by using first‐principles calculations that Mg_3_Bi_2_ is a spinless type‐II nodal‐line semimetal. The type‐II nodal loop lies around Γ, slightly above the Fermi level, and the drumhead surface states appear inside the tilted nodal loop. With the inclusion of spin–orbit coupling, only a small energy gap (≈35 meV) is opened at the line nodes, yielding an overall bulk band structure that still resembles that of a type‐II nodal‐line semimetal. The result is inconsistent with the work by Zhang et al., in which Mg_3_Bi_2_ with SOC is supposed to be a Dirac semimetal.^32^ To test this prediction, we have grown large high‐quality single crystals of this material and carried out angle‐resolved photoemission spectroscopy (ARPES) measurements. The ARPES results agree well with our first‐principles band calculations. These experiments and the stability of Mg_3_Bi_2_ under normal experimental conditions thus establish Mg_3_Bi_2_ as a promising material for experimentally studying exotic type‐II nodal‐line semimetals.

Mg_3_Bi_2_ crystalizes in a La_2_O_3_‐type structure in which Mg and Bi atoms form hexagonal layers and stack along the *c* direction as shown in **Figure** **1**a. The space group of the lattice is $P\bar{3}m1$ (No. 164) and the lattice is centrosymmetric. The crystal structure of our single‐crystal Mg_3_Bi_2_ samples was determined by X‐ray diffraction (XRD) measurements (see Figure 1b), confirming their identity. The first Brillouin zone of the trigonal lattice is shown in Figure 1c, with the high‐symmetry momentum points marked. The calculated bulk band structure of Mg_3_Bi_2_, obtained here using the generalized gradient approximation method without the inclusion of spin–orbit coupling, is shown in Figure 1d.

**Figure 1:**
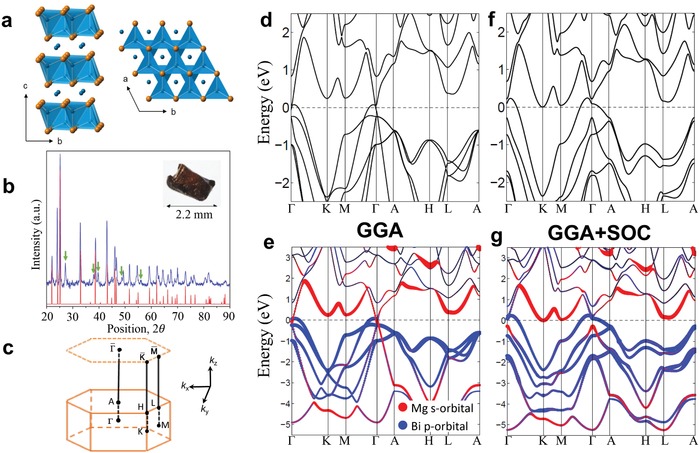
Crystal structure and bulk bands of Mg_3_Bi_2_. a) The lattice structure of Mg_3_Bi_2_; the blue and orange balls indicate the Mg and Bi atoms, respectively. b) Powder X‐ray diffraction (PXRD) pattern. The green arrows in the PXRD pattern correspond to the remaining Bi flux on the sample surface. c) Bulk Brillouin zone and projected (001)‐surface Brillouin zone. d) Bulk band structure without the inclusion of spin–orbit coupling. e) Bulk bands in part (d) with atomic orbital projection. f,g) Same as parts (d) and (e) but with the inclusion of spin–orbit coupling.

The low‐energy electronic behavior of this compound is seen to be predominantly determined by the dispersion of two bands in the vicinity of the Fermi level. The bands cross each other along the Γ–*K* and Γ–*M* directions and disperse apart in other directions, indicating a semimetallic property in this compound. The orbital projection (Figure 1e) indicates that the two bands originate from Bi 6p and Mg 3s orbitals. The band crossing in the Γ–*K* direction is gapless and the two bands around the crossing point possess the same sign for their slope, which is the hallmark of a type‐II Dirac point. Along Γ–*M*, by contrast, there exists a tiny gap between the two bands. Generally, in a spinless solid‐state system with both space‐inversion and time‐reversal symmetries, the node points must form a 1D line rather than discrete points, because the spinless Hamiltonian of two bands can be always taken to be real‐valued and the dimension of nodal solutions is 1 in 3D momentum space.^33^ Therefore, there must exist a nodal line passing the band crossing point in spinless Mg_3_Bi_2_. Our detailed band calculations demonstrate that the nodal points (NPs) indeed form a 1D loop surrounding Γ, and lie very close to the Γ–*M*–*K* plane. The nodal loop does not stay entirely within the Γ–*M*–*K* plane, but wiggles slightly with respect to the plane. This type‐II feature holds on every point of the nodal loop. Therefore, Mg_3_Bi_2_ is a type‐II nodal‐line semimetal in the spinless case. In the presence of spin–orbit coupling, an energy gap is opened everywhere along the nodal line and the materials become a strong topological insulator (see Figure 1f,g). The size of this spin–orbit gap is very small, ≈35 meV. Even though the type‐II nodal line is gapped in the calculation with SOC present, the two bulk bands around the gap still share the same sign of slope, suggestive of a similar low‐energy behavior of bulk carriers as the type‐II nodal‐line semimetals.

The critical distinction between the type‐I nodal line and the type‐II nodal line is the band dispersion along transverse directions of the nodal line. In the type‐I nodal‐line band structure, the two bands disperse with opposite slopes in both transverse directions, while in the type‐II nodal‐line band structure, the two bands are tilted in such a way that they share the same sign in their slope along one transverse direction. This is schematically plotted in **Figure** **2**a. The zoom‐in band structure of spinless Mg_3_Bi_2_ in Figure 2b clearly shows this characteristic of type‐II nodal lines. The two bands cross at a nodal point (orange dot point in Figure 2c) in the Γ–*K* direction (//*k_x_*) and form a tilted cone structure. Dispersing away from the NP point, the two bands have a quadratic touching along the tangent direction of the nodal line and form a normal cone along the other two transverse directions. We note that the band crossing is gaped along the Γ–*M* direction, so the nodal line is not entirely in the Γ–*M*–*K* plane. The 3D plot of the nodal line of Mg_3_Bi_2_ is shown in Figure 2c. The nodal line takes the form of a closed loop surrounding Γ point. The loop wiggles in the momentum space; thus, it does not stay in any momentum plane. This is so because the formation of this nodal loop is a general consequence of the presence of space‐inversion and time‐reversal symmetries. It does not rely on other crystalline symmetries, in contrast with the nodal lines in Ca_3_P_2_ 26 and PbTaSe_2_,^24^, ^29^ which are protected by the mirror reflection symmetry. To visualize the surface states, we calculated the band spectrum of a semi‐infinite Mg_3_Bi_2_ slab with a (001) surface. The surface can be terminated by either a Bi atomic layer (Figure 2d) or a Mg atomic layer (Figure 2e). In both cases, there exists a surface band inside the nodal line as highlighted in the insets of Figure 2d,e. This surface band connects to every bulk nodes along the nodal line, forming a 2D “drumhead” surface. The “drumhead” surface band is more dispersive on the Mg‐terminated surface.

**Figure 2:**
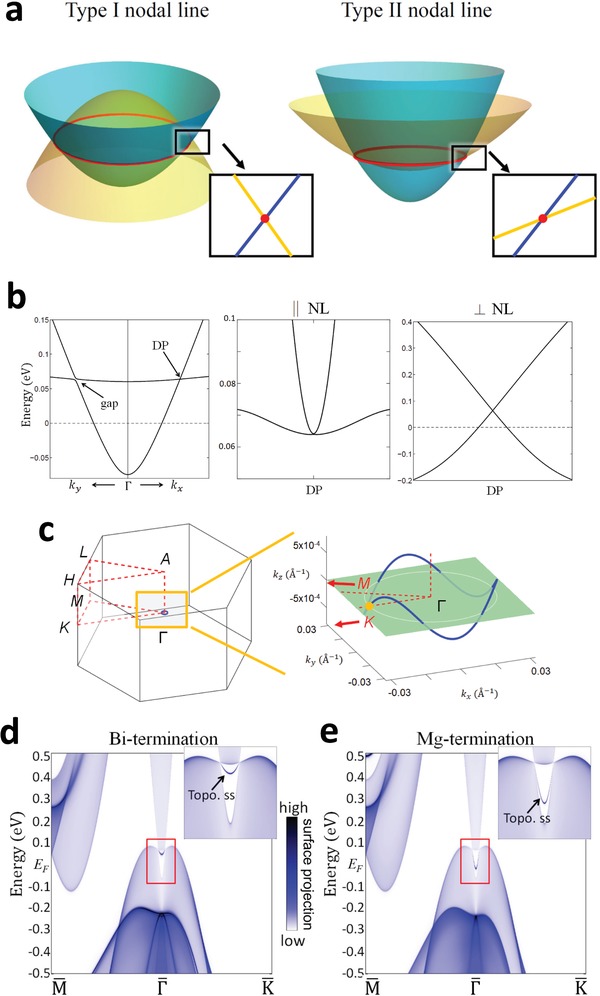
Type‐II nodal‐line band structure of Mg_3_Bi_2_ (without SOC). a) Schematic of type‐I and type‐II nodal‐line band structure. b) Bulk bands around a nodal point in the –Γ–*K* direction. Three panels show the band dispersion from the NP in three perpendicular (radial, tangential, and vertical) directions, respectively. c) The 3D plot of the nodal line of Mg_3_Bi_2_ with a closed loop surrounding the Γ point. d) Band spectrum of a semi‐infinite slab with a (001) surface. The surface is terminated at a Bi layer. The surface band is shown in the inset. The color indicates the weight of charge density of each state at the surface layer. e) Same as part (d), but for a Mg‐terminated semi‐infinite slab.

When SOC is included, the bulk nodal ring is gapped, which results in a continuous gap between the conduction and valence bands throughout the Brillouin zone. This result is inconsistent with previous theoretical work, which can be attributed to the different lattice constants employed in the calculation. The fully gapped state allows for a well‐defined *Z*_2_ topological invariant.^34^ We performed calculations with the Heyd–Scuseria–Ernzerhof (HSE) hybrid functional for an accurate estimate of the energy band gap. We calculate the *Z*_2_ topological invariant by the Wilson loop method (see **Figure** **3**a and Supporting Information).^35^ The Wilson band is an open curve traversing the entire Brillouin zone in the time‐reversal invariant plane *k_z_* = 0 and a closed loop in another time‐reversal invariant plane *k_z_* = π. The result indicates that the *Z*_2_ invariant equals 1, which indicates that Mg_3_Bi_2_ is a strong topological insulator in the presence of SOC.

**Figure 3:**
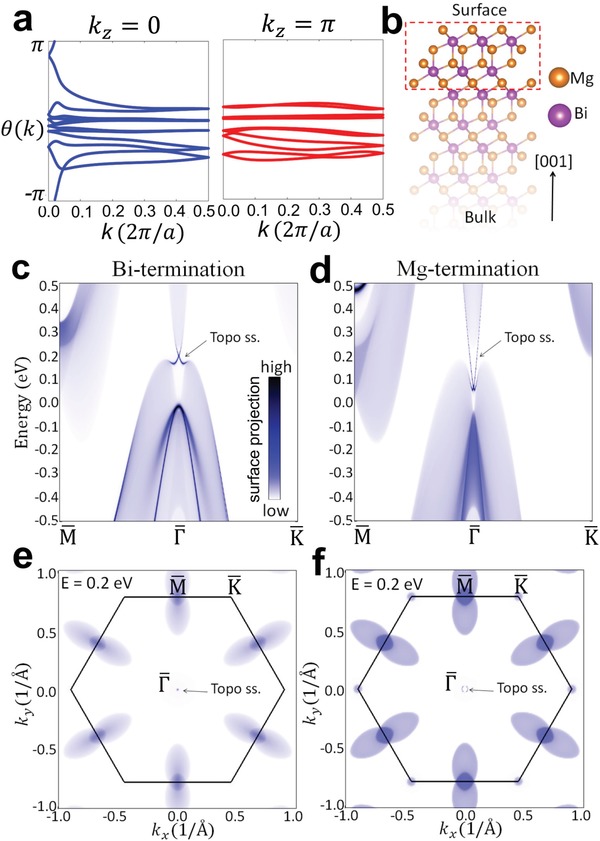
Topological invariant and surface band structure of Mg_3_Bi_2_ (with SOC plus HSE). a) Wannier charge center evolution in time‐reversal invariant planes. b) Lattice structure of a semi‐infinite slab. c) Band spectrum of a semi‐infinite slab with a Bi‐terminated (001) surface. The color indicates the weight of charge density of each state within the top six atomic layers. d) Same as part (c), but for a Mg‐terminated semi‐infinite slab. e,f) Calculated iso‐energy band contour at *E* = 0.2 eV for Bi‐terminated and Mg‐terminated semi‐infinite slabs, respectively.

The topological surface states are now calculated by way of the semi‐infinite Green's function. Each state is plotted with a color corresponding to the integrated charge density of the state within the top six atomic layers, depicted in Figure 3b. The thickness of the six atomic layers is about 10 Å, which roughly equals the typical escape depth of photoelectrons.^36^ Therefore, the weighted band spectrum of Mg_3_Bi_2_ (Figure 3c–f) can be directly compared to the ARPES results. For the Bi‐terminated surface, a topological Dirac surface band connects the conduction and valence bulk bands (Figure 3c). The Dirac point is about 0.2 eV above the Fermi level, inside the spin–orbit gap. Below the Fermi level, there exist a pair of surface resonance bands around Γ, which are buried inside the bulk band region. The topological surface band on the Mg‐terminated surface disperses deeper in energy with the Dirac point closer to the valence band edge (Figure 3d), unlike the Dirac point on the Bi‐terminated surface, which is closer to the conduction band edge. The drumhead surface states in the spinless case have a similar energy position with respect to the bulk band edges. This is no surprise because the drumhead surface band splits and transforms into topological Dirac surface states when SOC is turned on. Another distinction between the band spectra on Bi‐ and Mg‐terminated surfaces is that the resonance states on the Mg‐terminated surface are more diffusive into the bulk and thus the calculated band spectrum is less sharp compared to those of Bi‐terminated surface. The calculated iso‐energy band contours at *E* = 0.2 eV are shown in Figure 3e,f for Bi‐ and Mg‐terminated surfaces, respectively. This energy corresponds to that of the Dirac point on the Bi‐terminated surface. There are three prominent features in the band contour: a double lobe surrounding the $\bar{M}$ point, a little circular electron pocket centered at the $\bar{K}$ point, and a little loop from the topological surface states at the zone center. The loop is vanishingly small on the Bi‐terminated surface because the energy is set at the energy of the Dirac point.

To confirm the calculated band structure and thus the character of Mg_3_Bi_2_, we performed ARPES measurements on our single crystals. The spectrum taken along $\bar{K}\;-\;\bar{\Gamma }\;-\;\bar{K}$ with 74 eV photons is shown in **Figure** **4**a. The dominant feature in the spectrum is a hole‐like valence band pocket. Inside the bulk band region, there are three sets of surface states/resonances. The three sets of surface bands are all hole‐like, and the top of bands are at *E*_F_, −0.4 and −0.8 eV, respectively. All these band features are well reproduced by the electronic structure calculation for the Bi‐terminated semi‐infinite slab (Figure 4b), indicating that the measured cleaved sample surface is Bi‐terminated. We also carried out surface chemical doping to raise the Fermi level of the surface layers. Potassium deposition on the surface of Mg_3_Bi_2_ resulted in a Fermi level shift of 0.2 eV (Figure 4c), but further potassium deposition led to the deterioration of the spectra with no significant doping. The ARPES spectrum of the doped bands clearly shows the double humps at the top of the valence band above the Fermi level. The result, again, is consistent with the calculated band structure. The projected Fermi surface contour as shown in Figure 4d consists of a hexagonal pocket centered at $\bar{\Gamma }$ and a lobe close to$\;\bar{M}$. We note that the intensity of the lobe at $\bar{M}$ is weak because the states are bulk‐like with a very small fraction of charge distributed within the top atomic layers. There is a small circular contour inside the $\bar{\Gamma }$ pocket, which is from the surface resonance bands. This is in excellent agreement with our ARPES‐determined constant‐energy maps of Figure 4e. The $\bar{M}$ pocket is missing from the ARPES data most likely because of the weak spectral weight within the surface layers. The size of both the bulk band pocket and surface state pocket increases as the energy changes from 0 to −0.6 eV, indicative of the hole‐like nature of the bulk and surface bands.

**Figure 4:**
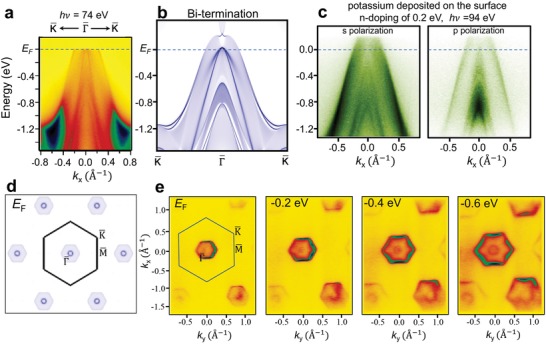
ARPES measurements on Mg_3_Bi_2_. a) ARPES spectrum taken along the $\bar{\Gamma }\;-\;\bar{K}$ direction. The photon energy is 74 eV. b) Calculated band spectrum of a semi‐infinite slab with a Bi‐terminated (001) surface. c) APRES spectra taken after potassium deposition of the surface. The two panels show the ARPES results taken with vertically and horizontally polarized photons, respectively. d) Calculated iso‐energy band contour at the Fermi level for a Bi‐terminated (001) surface. e) Iso‐energy ARPES mapping at different binding energies.

To establish the surface vs bulk nature of the measured states, we performed excitation energy‐dependent ARPES mapping (**Figure** **5**a). Using incident photons of 60–110 eV, corresponding to *k_z_* values of 4.2–5.5 Å^−1^, we show that the four inner sharp bands (marked by the four dashed lines in Figure 5a) have no *k_z_* dependence. This indicates the surface nature of those states, and is consistent with the theoretical band structure shown in Figure 3c. The outer, broader bands have only weak *k_z_* dependence, but according to our calculations they can only be ascribed to low‐dispersing bulk states localized within the quadruple layers of Figure 1a. The two surface resonance bands form concentric circles in the iso‐energy contour at the Fermi level as shown in Figure 5b. This double‐circle surface Fermi surface is well reproduced in the first‐principles calculations (Figure 5b). The topological character of Dirac surface states is further corroborated by inspecting the calculated spin texture of the surface band, which is shown in Figure 5c–f. The band spectra are plotted along $\bar{K}\;-\;\bar{\Gamma }\;-\;\bar{K}$ (//*k_x_*) for the Bi surface termination (Figure 5c) and the Mg surface termination (Figure 5d), and the corresponding spin textures for the two possible surface terminations are reported in Figure 5e,f, respectively. The calculated dominant spin component of the topological surface states is 〈*S_y_*〉; the other two spin components are found to be negligible. Therefore, the calculated spin polarization of the Dirac surface states is perpendicular to the direction of the momentum, and thus exhibits the spin–momentum locking configuration that is characteristic of topological surface states. The surface resonance bands also exhibit a similar helical spin texture with a dominant spin component 〈*S_y_*〉. The two surface resonance bands on the Bi‐terminated surface share the same spin helicity. This indicates that although the two bands share the same vertex point, they actually do not form a Rashba‐type band structure. (The two branches of a Rashba spin‐split band should have opposite spin helicities).^37^, ^38^

**Figure 5:**
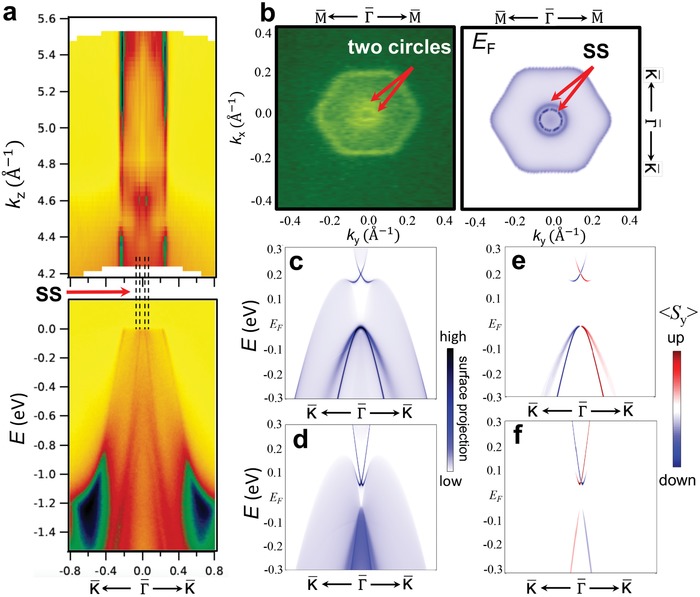
ARPES characterization of the surface states of Mg_3_Bi_2_. a) Momentum distribution curves at the Fermi level taken with different photon energies (and thus different *k*_z_ values). b) ARPES and calculated iso‐energy contours at the Fermi level. The two inner circles are the surface states. c,d) Calculated band spectra along the $\bar{\Gamma }\;-\;\bar{K}$ direction for Bi‐terminated and Mg‐terminated semi‐infinite slabs, respectively. The color indicates the surface weight of each state. e,f) Calculated spin texture for Bi‐terminated and Mg‐terminated semi‐infinite slabs, respectively. The color indicates the spin polarization along the *S_y_* direction.

We initially investigated the electronic band structure of Mg_3_Bi_2_ by first‐principles calculations, motivated by the possibility that it might have topological electronic states. Single‐crystal Mg_3_Bi_2_ samples were then grown and characterized by ARPES to test our prediction. The remarkable congruence of the ARPES measurements and the first‐principles calculations unambiguously confirms the electronic band structure of Mg_3_Bi_2_. We find that Mg_3_Bi_2_ is a spinless type‐II nodal‐line semimetal. The tilted nodal line is a closed loop centered at the Γ point and the drumhead surface band is found inside the loop of bulk nodes. The spin–orbit interaction opens a band gap ∆*E* of ≈35 meV at the bulk line nodes and makes the compound a strong topological insulator. The Dirac surface states in the topological insulator phase are derived from the drumhead surface states by incorporating SOC. It is important to note that the energy gap of Mg_3_Bi_2_ is comparable to or *smaller* than the spin–orbit gap of typical type‐I nodal‐line compounds such as Cu_3_PdN (∆*E* = 62 meV).^27^, ^28^ The SOC present in Mg_3_Bi_2_ is mainly the result of the strong atomic SOC of Bi. Therefore, the very small spin–orbit band gap can be reduced in future work by substitutional alloying, for example, by partially replacing Bi by Sb or As and making an Mg_3_Bi_2−_ *_x_*Sb(As)*_x_* alloy, a method that has been experimentally proven as effective in tuning the band gap of topological materials.^38^ Detailed simulation and experimental tests of the effect of doping in this material are left as an open question for future studies. Our theoretical and experimental results show that Mg_3_Bi_2_ is the archetype of a promising new family of topological semimetals for studying the exotic properties of type‐II nodal‐line fermions and the associated drumhead surface states—a new topological electronic system that has thus far been inaccessible to experimental study.

## Experimental Section

Mg_3_Bi_2_ single crystals were grown out of a Bi‐rich solution. Starting elements were loaded in alumina Canfield Crucible Sets^39^ with a molar ratio of Mg:Bi = 15:85, and sealed in a silica tube under partial argon atmosphere. The ampoule was then heated up to 600 °C and slowly cooled to 300 °C, at which temperature the crystals were separated from the solution in a centrifuge. Samples were analyzed for phase identification and purity using a Bruker D8 powder X‐ray diffractometer equipped with Cu Kα radiation (λ = 1.5406 Å). According to the XRD pattern shown in Figure 1b, Mg_3_Bi_2_ was successfully grown, as the majority of the reflections were indexed according to the reported La_2_O_3_‐type structure. Single‐crystal X‐ray diffraction (Bruker Apex II diffractometer Mo Kα1 radiation) confirmed the identity and high quality of the Mg_3_Bi_2_ crystals grown.

Photoemission data were collected at Advanced Light Source beamline 4.0.3 employing linearly polarized 60–110 eV photons, Scienta R8000 analyzer in dithered fixed mode, ±15° angular lens mode, and polar angle scanning in steps of 1/3° for the perpendicular direction. The sample was attached to the cryostat, cleaved in ultrahigh vacuum at 35 K, and measured within 8 h, showing linewidths of less than 0.025 Å^−1^.

The electronic structures were computed using the projector augmented wave method^40^, ^41^ as implemented in the VASP package^42^ within the generalized gradient approximation scheme.^43^ The SOC was included self‐consistently in the calculations of electronic structures with a Monkhorst–Pack *k*‐point mesh 15 × 15 × 11. To calculate the surface electronic structures, a first‐principles tight‐binding model Hamilton was constructed by projecting onto the Wannier orbitals,^44^, ^45^, ^46^ which use the VASP2WANNIER90 interface.^47^ Mg s and p orbitals and Bi p orbitals were used to construct Wannier functions and the procedure for maximizing localization was performed. The surface states were calculated from the surface Green's function of the semi‐infinite system.

## Conflict of Interest

The authors declare no conflict of interest.

## Supporting information

Supplementary
