# Supplementary material for: Realization of a Type‐II Nodal‐Line Semimetal in Mg3Bi2
Source: Adv Sci (Weinh). 2018 Nov 28;6(4):1800897. doi: 10.1002/advs.201800897 (PMC6382304; doi:10.1002/advs.201800897)
Supplement: Supplementary file 1 — Supplementary [file ADVS-6-1800897-s001.pdf]

Copyright WILEY-VCH Verlag GmbH & Co. KGaA, 69469 Weinheim, Germany, 2018.

ADVANCED SCIENCE Open Access

Supporting Information

for *Adv. Sci.,* DOI: 10.1002/advs.201800897

Realization of a Type-II Nodal-Line Semimetal in Mg_3Bi_2

Tay-Rong Chang, Ivo Pletikosic, Tai Kong, Guang Bian, Angus Huang, Jonathan Denlinger, Satya K. Kushwaha, Boris Sinkovic, Horny-Tay Jeng, Tonica Valla, Weiwei Xie, and Robert J. Cava*

Supporting Information

Realization of a Type-II Nodal-Line Semimetal in Mg_3Bi_2

Tay-Rong Chang,[1] Ivo Pletikosic,[2] Tai Kong,[3] Guang Bian,[4] Angus Huang,[5] Jonathan Denlinger,[6] Satya K. Kushwaha,[3] Boris Sinkovic,[7] Horny-Tay Jeng,[5,8,9] Tonica Valla,[10] Weiwei Xie,[11] Robert J. Cava[2]

Z_2 topological phase of Mg_3Bi_2 with spin-orbit coupling

According to band theory, Mg_3Bi_2 is not an "insulator" since there is no global energy gap. However, the band topology is not determined by the band gap size. In condensed matter physics, the band topology is identical for two different systems as long as the band structure of one system can be continously transformed to that of the other system without closing the band gap. Therefore, if the valence bands are separated from conduction bands by a "continuous gap" throughout the whole BZ, the Z_2 topological invariant can be well-defined [1,2] (in the same way as topological insulators). The Wilson loop of k_x, k_y, and k_z (Figure S1 and Figure 3a) indicate that the Z_2 invariant equals 1, so Mg_3Bi_2 is equivalent to a topological insulator in the band structure. This is why Mg_3Bi_2 is described as a topological insulator in the presence of SOC in this work, despite the lack of a global energy gap.

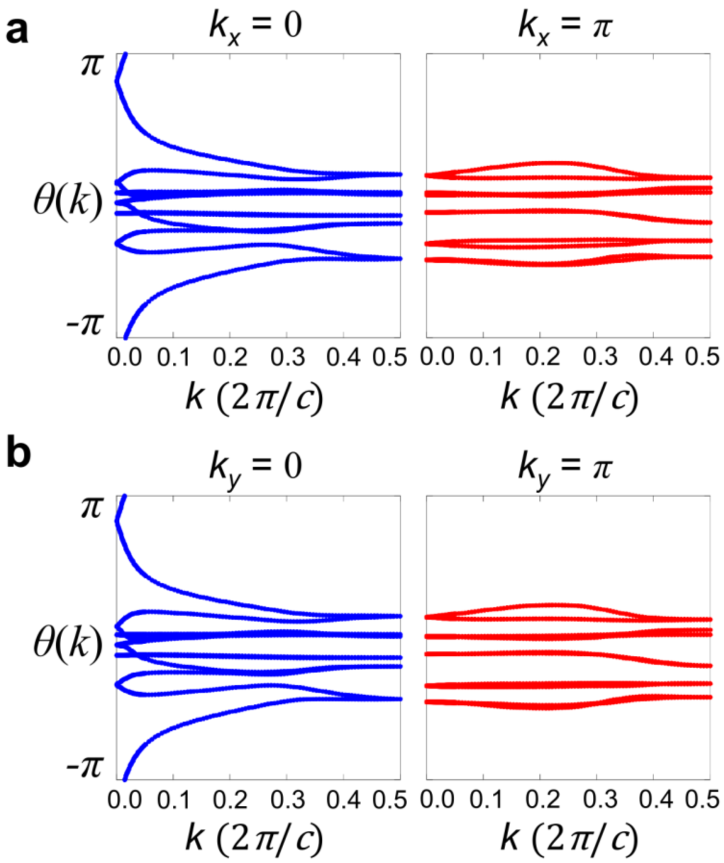

Figure S1. Topological invariant and surface band structure of Mg_3Bi_2 (with SOC plus HSE). a) Wannier charge center evolution in the time-reversal invariant planes at k_x = 0 and π. b) Same as a), but for the time-reversal invariant planes at k_y = 0 and π.

Band dispersions on nodal-line at $k_z \neq 0$

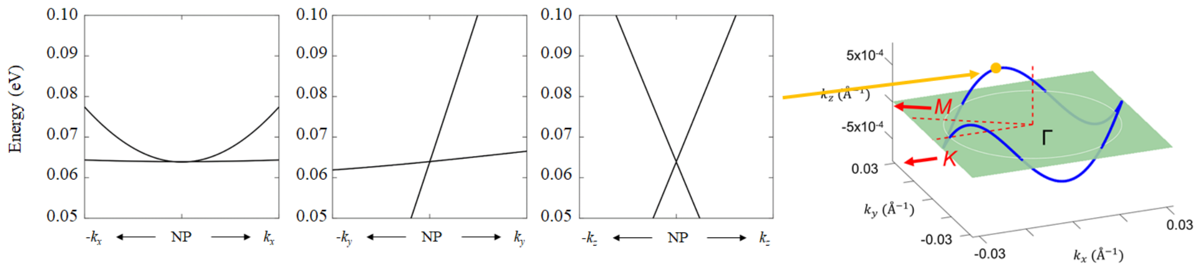

Figure S2. Band dispersion close to the point marked by the orange ball ($k_z \neq 0$) in three perpendicular (tangential, radial, and vertical) directions.

[1] M. N. Ali, Q. D. Gibson, T. Klimczuk, and R. J. Cava , Phys. Rev. B 89, 020505(R) (2014).
[2] T.-R. Chang, P.-J. Chen, G. Bian, S.-M. Huang, H. Zheng, T. Neupert, R. Sankar, S.-Y. Xu, I. Belopolski, G. Chang, B. Wang, F. Chou, A. Bansil, H.-T. Jeng, H. Lin, and M. Z. Hasan , Phys. Rev. B 93, 245130 (2016).
